# Finite element analysis of progressive collapse resistance of a prefabricated RC frame structure with stud connection considering chloride corrosion

**DOI:** 10.1371/journal.pone.0333741

**Published:** 2025-11-20

**Authors:** Zhiyuan Gao, Jiaolei Zhang

**Affiliations:** 1 School of Civil and Transportation Engineering, Yellow River Conservancy Technical University, Kaifeng, China; 2 College of Urban Development and Modern Transportation, Xi’an University of Architecture and Technology, Xi’an, China; Universiti Teknologi Malaysia, MALAYSIA

## Abstract

To evaluate and ensure the safety of a prefabricated connection scheme for future engineering applications, this study investigates the life-cycle progressive collapse resistance of prefabricated reinforced concrete frame structures (PRCS) with steel tube stud connections. A simplified prefabricated column model was developed in SAP2000 using multi-segment linear plastic connection elements, and a six-story PRCS was established for analysis. Progressive collapse behavior of the PRCS and a comparable cast-in-place concrete frame structure (CPCS) was evaluated through the column removal method under four scenarios: corner column, long-side middle column, short-side middle column, and interior column removal. Time-history responses of internal forces and displacements were obtained. In addition, the effect of chloride-induced corrosion on the collapse performance of the structure was analyzed through pushdown analyses. The results indicate that the proposed connection model accurately reproduces the behavior of prefabricated column joints. Among the four scenarios, interior column removal had the most severe effect on PRCS, with displacement increasing by 72.2% compared to CPCS. Pushdown analysis revealed that PRCS exhibited 25.4% lower beam mechanism capacity when the corner column was removed, and 33.1% lower catenary mechanism capacity when the interior column was removed. Furthermore, long-term corrosion significantly reduced the progressive collapse capacity, underscoring the necessity of considering durability effects in design.

## 1 Introduction

The progressive collapse of the structure is usually caused by accidents or accidental loads, which triggers the domino effect to aggravate the diffusion of damage. Finally, the whole structure loses its bearing capacity and then collapses in a large area [1]. The progressive collapse of building structures usually causes significant casualties and economic losses. Therefore, it is of great significance to study the progressive collapse performance of building structures. For prefabricated building structures, such structures are an important way for countries around the world to achieve building industrialization, because prefabricated structures have the characteristics of high construction efficiency, environmental protection, and integrated production [2]. At the same time, the prefabricated structure is also the carrier of the current intelligent construction implementation, and plays an important role in promoting the implementation of carbon neutrality. However, previous studies usually focus on the seismic performance of the joints of the prefabricated structure, and less on the collapse resistance of the joints of the prefabricated in the frame structure. For example, on March 7, 2020, a prefabricated frame structure in Quanzhou, China, suddenly collapsed during long-term service, causing a large number of casualties [3]. Although the problem of progressive collapse has been widely emphasized by engineers, most of the current progressive collapse specifications are for CPCS, and there is less guidance for the progressive collapse design of PRCS, and the damage effect of long-term chloride corrosion on the structure is usually ignored. A large number of experiments have shown that long-term chloride corrosion will lead to a significant decrease in the mechanical properties of prefabricated joints [4].

Since the collapse of the 18-story prefabricated structure of the Ronan Point apartment in the United Kingdom due to a local explosion and the progressive collapse of the World Trade Center in New York [5,6], many scholars have recognized the importance of progressive collapse design of building structures. Therefore, in recent years, some scholars have gradually carried out research on the quasi-static test of prefabricated columns and the progressive collapse resistance of structures. Meanwhile, other studies have highlighted that current progressive collapse analysis approaches need to account for dynamic effects such as strain-rate sensitivity and Dynamic Increase Factor (DIF), since the sudden removal of a column is essentially a dynamic phenomenon. Ferraioli [7] emphasized the role of strain-rate effects in column removal scenarios, while Ferraioli [8] demonstrated that the DIF should be calibrated with ductility demand and structural configuration rather than assuming a fixed amplification factor. However, these studies have predominantly focused on CPCS, and systematic investigations for prefabricated reinforced concrete structures (PRCS) remain limited. In the present study, DIF is not explicitly considered, which is a limitation to be addressed in future research. For example, Zhang et al.[9] also proposed a prefabricated column with self-tapping stud connection. Experimental results indicate that failure initiated at the bottom of the column foot, while the studs remained intact. Yang et al. [10] proposed a self-centering prefabricated column using studs and prestressed steel bars. Through static tests, it was found that the energy dissipation of the structure was superior. In addition, Kang et al. [11] also conducted a progressive collapse test on a composite beam and a prefabricated column. By removing the corresponding column members in turn, the reinforcement ratio, bottom reinforcement structure and composite surface structure were studied. The results show that after removing the relevant key components, the prefabricated structure has arch effect and catenary effect. Finally, the longitudinal reinforcement of the beam is pulled out, which leads to the final failure of the structure. At the same time, the performance suggestions for improving the structural resistance are given. Al-Salloum et al. [12] carried out a static Pushdown test on the prefabricated concrete substructure connected by the bracket bolt rod. It was found that the progressive collapse resistance of the structure connected by the bracket bolt rod was low. The bearing capacity of the structure after reinforcement with steel plates and bolts was significantly improved, but the ductility of the joints was insufficient under large deformation, which led to brittle shear failure of the structure. Qian et al. [13] four 1:3 scale prefabricated beam-column structures were completed, and the progressive collapse test of the central column was carried out. Four specimens involved different connection modes and cast-in-place joints for comparative analysis. The results show that the progressive collapse effect of wet connection is better than that of cast-in-place joints, and the dry connection has fast damage degradation due to the stress concentration of bolts. Nimse et al. [14] four kinds of wet connections, such as mechanical sleeve, bending anchorage and prestressed connection, and a cast-in-place specimen were studied. The results show that the sleeve connection failure occurs at the sleeve connection. When the bending anchorage of the beam is replaced, the collapse resistance performance reaches the same level as that of the cast-in-place. At the same time, the pre-stress can significantly improve the collapse resistance of the two.

However, the above studies ignore the effect of long-term corrosion damage on the progressive collapse performance of structures. Because some scholars have found that corrosion damage will reduce the progressive collapse resistance of the structure. For example, Qin et.[15] analyzed the progressive collapse performance of cast-in-place RC frame structures by considering the corrosion of reinforcement, and the results show that the corrosion of reinforcement will reduce the structure’s catenary and membrane effect. By considering the influence of chloride corrosion, Bao et al. [16] found that corrosion damage can lead to the ultimate bearing capacity of cast-in-place RC frame structure reduced by up to 43.8%. It is worth noting that these studies focus more on the effect of corrosion damage on the progressive collapse performance of cast-in-place frame structures. Although the above tests have also conducted a preliminary study on the progressive collapse resistance of PRCS [13–14], it is still necessary to further test the mechanical performance of column joints under large collapse deformation. At the same time, it is necessary to consider the influence of long-term corrosion damage on the progressive collapse resistance of PRCS, so as to reveal the progressive collapse resistance mechanism of PRCS more deeply and comprehensively.

**Fig 1:**
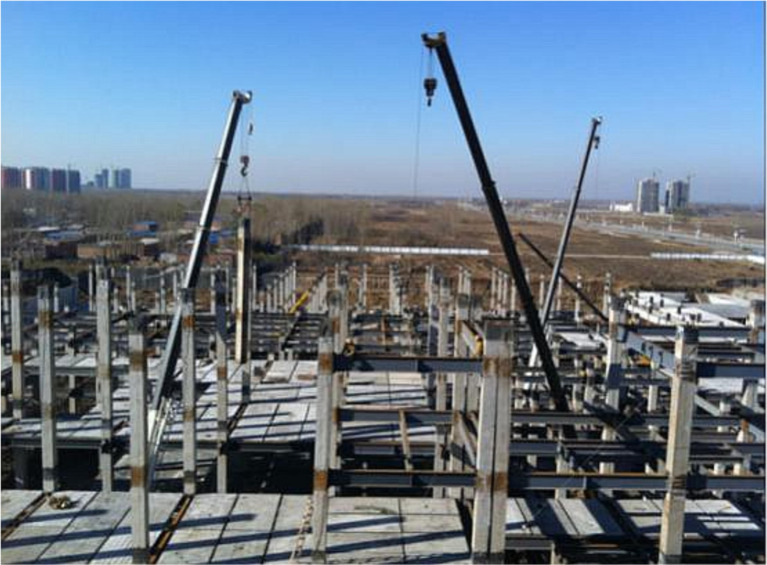
The application of prefabricated columns in practical engineering [20].

It should be noted that the aforementioned studies on the progressive collapse of PRCS have predominantly employed solid-element simulation models, which are computationally intensive and time-consuming. In life-cycle analyses of PRCS, multiple time points need to be considered to account for the effects of varying corrosion damage on the structure’s progressive collapse performance. To address this, this study models prefabricated columns with partially encased steel tube stud connections using multi-segment plastic connection elements to investigate their progressive collapse behavior. To address this, this study employs SAP2000 as the finite element analysis platform and models prefabricated columns with partially encased steel tube stud connections using multi-segment plastic connection elements to investigate their progressive collapse behavior. SAP2000 was chosen due to its user-friendly interface, which facilitates understanding and application of the results by engineers. Moreover, a considerable number of previous studies have employed SAP2000 to investigate progressive collapse and nonlinear structural behavior, demonstrating its reliability and accuracy in capturing plastic hinge development, catenary action, and component failure processes [17,18]. The seismic performance of this prefabricated column has been fully studied and can reach the same level as cast-in-place [19]. This component is easy to install, and the bending and shear bearing capacity of the joint meets the requirements. The energy dissipation and ductility of the component are excellent [20], which has been applied to practical engineering (Fig 1). However, the overall collapse resistance of the PRCS has not been systematically discussed. Based on this, this paper will remove the key components of a 6-story PRCS and a 6-story CPCS in turn, analyze and compare the corresponding internal force, displacement time history and structural Pushdown curve of the two, and consider the effect of chloride corrosion on the progressive collapse resistance of PRCS during the life cycle.

## 2 Introduction of basic information of prefabricated column

This section will introduce the mechanical performance and some design details of prefabricated columns with stud connections. The cross-section of the prefabricated columns was 400 mm and 400 mm, and the longitudinal reinforcement of the columns was also 8 HRB400 rebars with a diameter of 22 mm. The stirrups were all high-strength composite spiral stirrups provided by the China Academy of Building Research. The diameter of the stirrup was 5 mm, the yield strength of the stirrup was 1050 MPa, and the stirrup spacing was 50 mm. The stirrup encryption area was set at the height of 400 mm at the top of the column. The spacing is 30 mm, and the column height is 2100 mm. The concrete grade is C40, and the design value and standard value of concrete compressive strength are 29.27 MPa and 43.76 MPa respectively. The upper column and the lower column are disconnected, and the longitudinal reinforcement is discontinuous. The outer steel pipe is used at the connection, the transverse stud is penetrated, and the transverse stud is welded on the steel pipe. The steel pipe material is Q235 grade, the thickness is 5 mm. The stud is HRB400 rebar with a diameter of 22 mm.

**Fig 2:**
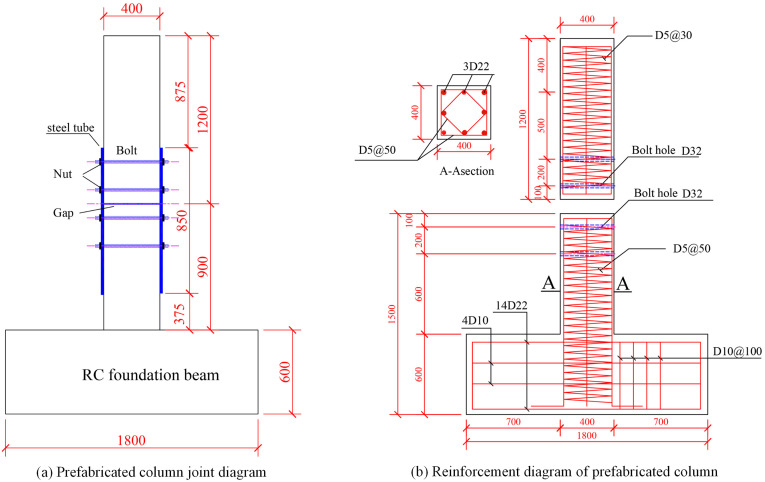
Joint design diagram of prefabricated column [20,21]. **(a)** Prefabricated column joint diagram. **(b)** Reinforcement diagram of prefabricated column.

In the authors’ previous study [19–21], the seismic performance of three prefabricated columns PRCC-W02, PRCC-W03, PRCC-W04 and one cast-in-place column W01 were investigated respectively. The detailed design parameters are shown in Table 1. The strength of the materials is shown in Table 2. The detailed structure of the prefabricated column is shown in Fig 2.

**Table 1:** Design parameters of prefabricated column.

Specimen number	Steel thickness	Axial compression ratio	Shear span ratio	Volume stirrup ratio/%
W01	—	0.60	4.75	0.79
PRCC-W02	5	0.35	4.75	0.79
PRCC-W03	8	0.60	4.75	0.79
PRCC-W04	5	0.60	4.75	0.79

**Table 2:** Basic mechanical properties of materials.

Materials	Reinforcement	Stirrup	Steel
Yield strength/MPa	490	1050	320
Ultimate strength/MPa	662.5	1170	475

The yield load, yield displacement, ultimate load, ultimate displacement, and equivalent viscous damping coefficient (ζ) [20] before yielding for each specimen after loading are summarized in Table 3. It can be observed that the mechanical performance of the prefabricated columns is generally comparable to those of the cast-in-place columns. Moreover, the prefabricated columns exhibit larger ultimate displacements, indicating better ductility. Therefore, to further ensure structural safety in engineering applications, this study proceeds to analyze the progressive collapse performance of a frame structure incorporating these prefabricated columns.

**Table 3:** Specimen in different stages of bearing capacity and displacement value [20].

Specimen No.	Yield point		Peak point		ultimate point		*ζ*
	*P*_y_/Kn	*D*_y_/mm	*P*_y_/Kn	*D*_y_/mm	*P*_y_/Kn	*D*_y_/mm	
W01	249.59	15.44	296.8	23.57	252.28	47.38	0.10
	−281.45	−16.33	−326.58	−23.24	−277.59	−47.39	
PRCC-W02	204.83	15.63	249.42	37.33	212.01	72.09	0.11
	−227.89	−14.09	−262.81	−34.48	−223.39	−84.92	
PRCC-W03	268.12	18.12	325.25	33.37	276.49	56.36	0.07
	−248.76	−13.61	−289.9	−21.91	−246.16	−56.36	
PRCC-W04	253.98	17.83	302.17	29.60	256.84	55.26	0.11
	−260.91	−16.13	−307.21	−28.91	−261.13	−70.15	

## 3 Steps of removing column method and establishment of structural model

### 3.1 Progressive collapse analysis based on removing column method

The removing column method, also known as the alternate load path method (removal component method), removes the main stress components in turn according to the corresponding requirements, checks the resistance of the remaining structure, and studies the internal force changes of the main stress components. The removing column method does not depend on the form of load on the structure, and pays more attention to whether the design performance of the structure itself is reasonable, and whether it is reasonable to select the initial damage component.

**Fig 3:**
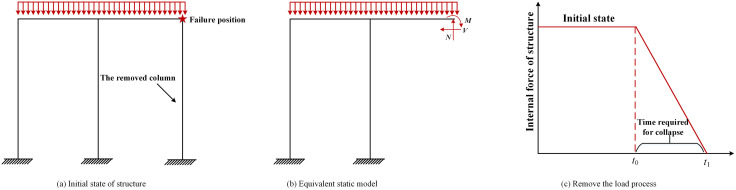
Schematic flow of the removing column method. **(a)** Initial state of structure. **(b)** Equivalent static model. **(c)** Remove the load process.

The specific implementation steps of the removing column method are as follows: 1) Determine the column members that need to be removed, as shown in Fig 3(a). 2) The static analysis of the structure to be analyzed is carried out, and then the internal forces (bending moment, shear force, axial force, etc.) of the column members to be removed are obtained, as shown in Fig 3(b). 3) Before removing the target column members, the dead and live loads are gradually applied until reaching a stable state. These loads are then held constant during an additional waiting time *t*_w_ to minimize dynamic effects. 4) After the relevant internal forces are obtained, the target column members are removed, and the above-mentioned internal forces are applied to establish an equivalent static model, as shown in Fig 3(b). 5) The applied internal force is removed according to the requirements of Code for Design of Building Structures against Collapse (CECS392–2014) [22]. The failure time from *t*_0_ to *t*_1_ shall not exceed 1/10 of the vertical basic period of the structure after the removal of the column members, as shown in Fig 3(c). Both geometric and material nonlinearities are fully considered in the progressive collapse analysis.

### 3.2 Establishment of simplified model of column-column joints

**Fig 4:**
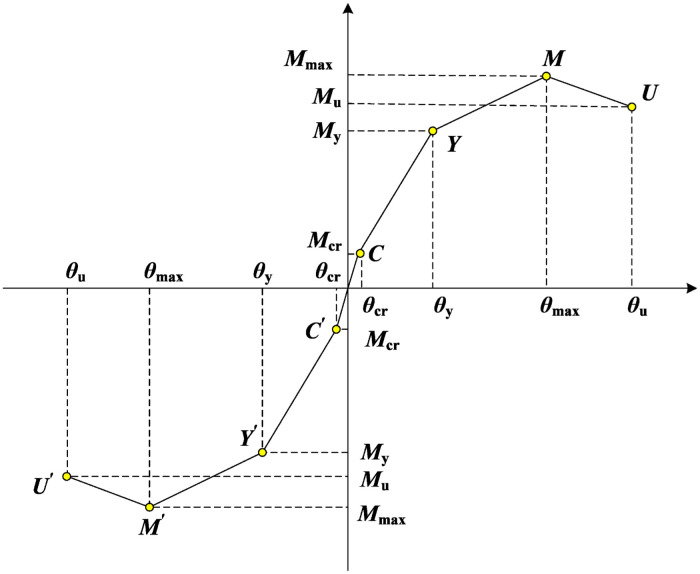
Stiffness values of prefabricated column joints in different failure stages.

**Fig 5:**
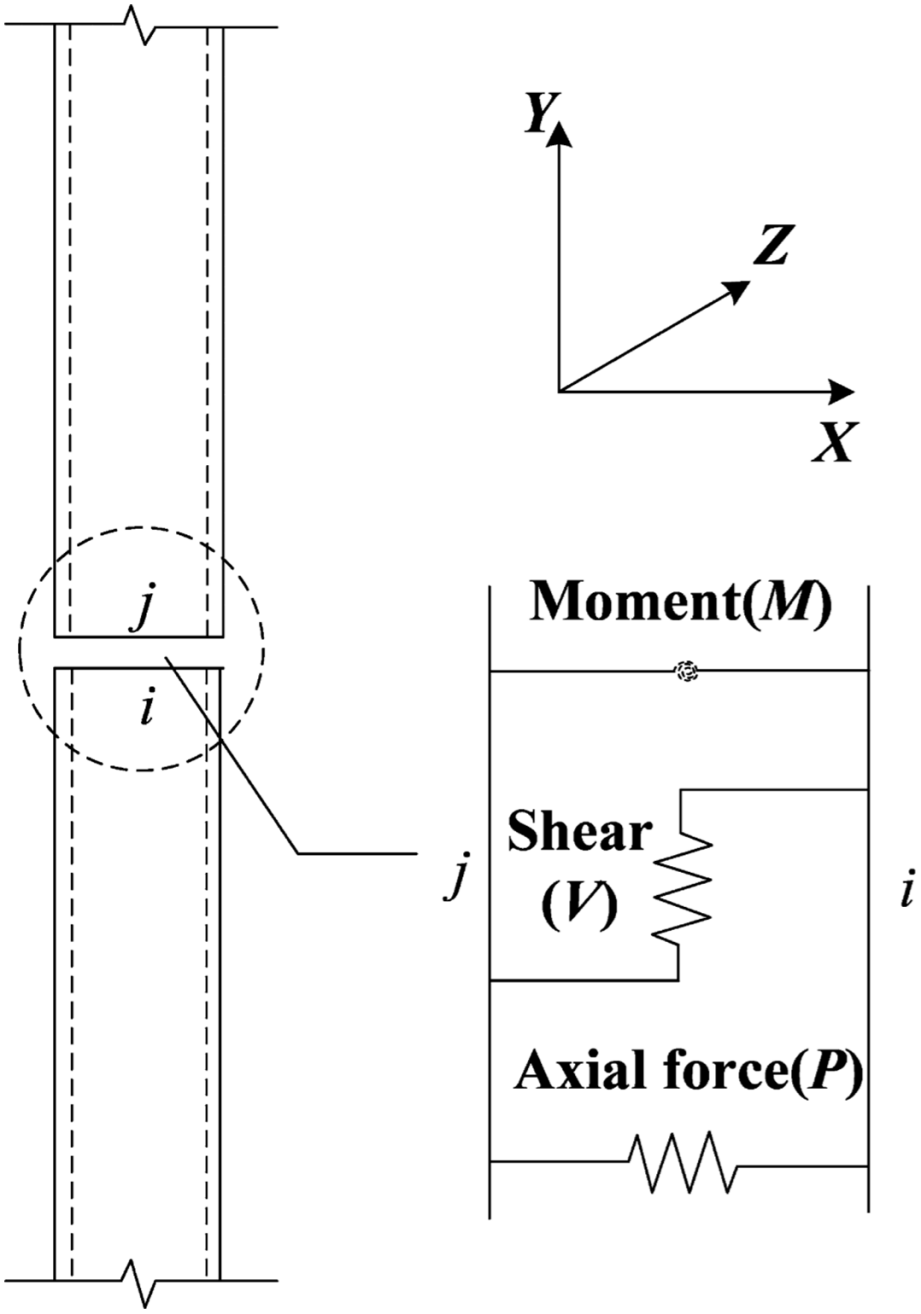
Schematic diagram of the prefabricated joint model.

It is difficult to converge and very time-consuming to calculate PRCS using traditional solid elements [23]. Therefore, based on SAP2000 V21.0 finite element software, a simplified model of prefabricated column based on multi-segment linear plastic connection elements is proposed in this paper. The simplified model releases the rotational stiffness of the prefabricated column and inputs the rotational stiffness of the prefabricated column joint at different stress stages in the *R*_1_ and *R*_2_ rotation directions of the nonlinear connection elements to achieve its stiffness equivalence, as shown in Fig 4. The feature points include crack point *C*(*θ*_cr_, *M*_cr_), yield point *Y*(*θ*_y_, *M*_y_), peak point *M*(*θ*_max_, *M*_max_) and ultimate point *U*(*θ*_u_, *M*_u_). The *K*_1_, *K*_2_, *K*_3_, *K*_4_ are the stiffnesses at different stages, respectively. The connecting element is composed of six decoupled springs, which are three springs in the *XOY* plane, which constrain the axial deformation, shear deformation and bending deformation respectively. Among them, the point *i* is in the lower column and point *j* is in the upper column, where the rotational degree of freedom of the *ij* node is released, and its translational degree of freedom is constrained, as shown in Fig 5. The column adopts the frame element, the rebar constitutive model adopts the two-fold line strengthening model, the concrete constitutive model is calculated by the Mander model [19], and the hysteresis curve type is selected by the Takeda model [24].

**Fig 6:**
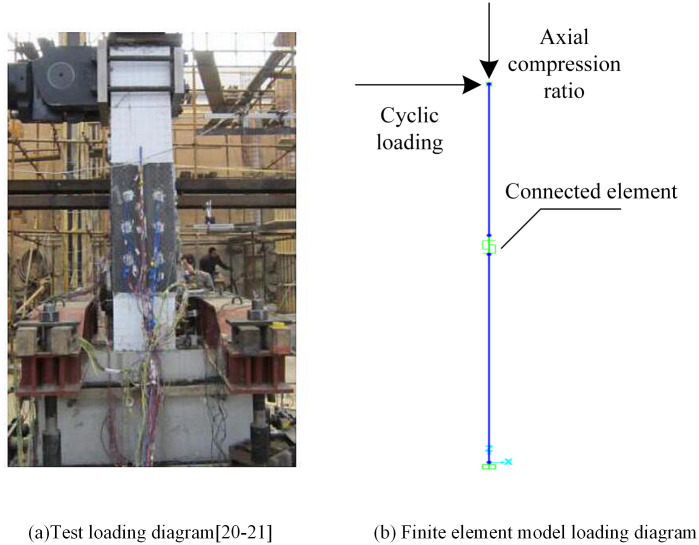
Loading diagram of test. **(a)** Test loading [20,21]. **(b)** Finite element model loading diagram.

**Fig 7:**
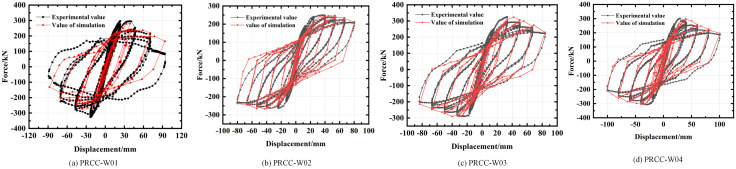
Column-column joint hysteretic curve verification [20,21]. **(a)** PRCC-W01. **(b)** PRCC-W02. **(c)** PRCC-W03. **(d)** PRCC-W04.

To validate the accuracy of the proposed simplified model, quasi-static cyclic tests were conducted on three prefabricated column joints, and the modeling approach described above was applied to simulate the test responses. Compared to monotonic pushdown loading, quasi-static cyclic tests offer a more comprehensive depiction of the nonlinear behavior of structural components, particularly in terms of strength degradation, energy dissipation, and stiffness deterioration. The envelope curve derived from quasi-static hysteresis responses inherently represents the monotonic backbone behavior of the component. According to ASCE 41, cyclic envelope and monotonic pushdown curves are comparable, as both capture key strength–deformation states. Therefore, the use of the envelope of hysteresis curves as an equivalent input for component-level collapse assessment is widely accepted [25]. The test and finite element loading are shown in Fig 6, and the calculation results are compared with the test results in Fig 7. The comparison shows that the simulation results, including the envelope curves, are in good agreement with the experimental data, demonstrating the accuracy and reliability of the proposed simplified model. Finally, the multi-segment linear plastic connection element is arranged at the column foot of each column of the frame structure to consider its most unfavorable situation.

From Fig 7, it can be seen that the simulated hysteresis curve is in good agreement with the experimental value, indicating that the multi-segment linear plastic connection element used in this paper can better simulate the prefabricated column joints, which provides a basis for the establishment of the PRCS frame structure model below. The average error between the peak bearing capacity based on the simplified model simulation and the test results is about 6.8%. This error is mainly due to the simplified model in order to achieve efficient calculation, ignoring the bond slip of reinforcement, the contact between reinforcement and concrete. Previous studies indicate that under monotonic loading, joints may behave slightly differently, and further investigation is needed in the future to assess the model’s accuracy under such conditions [26,27].

### 3.3 The establishment of frame structure model

**Fig 8:**
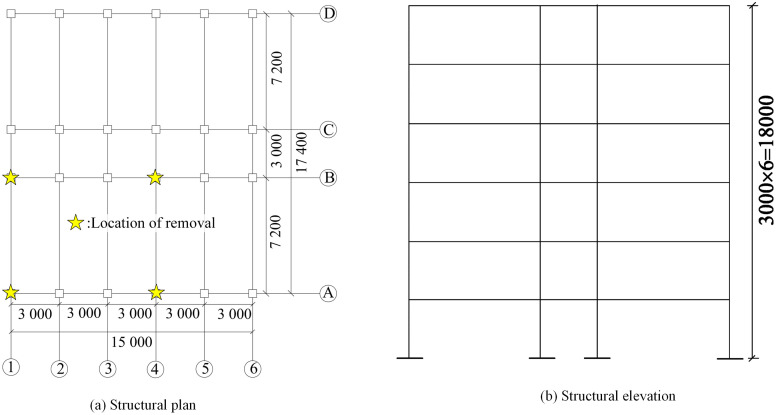
Six story PRCS and CPCS diagram. **(a)** Structural plan. **(b)** Structural elevation.

According to the Chinese code for structural design of buildings [28,29], a six-story PRCS is designed. The reinforcement and size of CPCS are the same as PRCS. The seismic grouping of the structure is Group II, with a site characteristic period of 0.4 second. Structures are located in category II sites. The seismic intensity of the structure is 8 degrees and the structural importance factor is 1.0. The basic size of the structure is shown in Fig 8. The cross-sectional dimensions of the columns are 500 mm and 500 mm, and the cross-sectional dimensions of the beams are 300 mm and 600 mm, 200 mm and 400 mm, respectively. The floor adopts membrane element, and the thickness of the floor is 250 mm. Other beam-column members adopt frame element. The concrete constitutive adopts Mander constraint relationship, and the rebar adopts two-fold line strengthening constitutive. The roof dead load is 5 kN/m^2^, the roof live load is 0.5 kN/m^2^, the floor dead load is 4 kN/m^2^, the floor live load is 2 kN/m^2^, the beam line load is 9.6 kN/m, the basic wind pressure is 0.35 kN/m^2^, and the basic snow pressure is 0.25 kN/m^2^. The member removal rules are based on the GSA 2013 [30] recommendations for removal, and four main collapse conditions are analyzed. 1) Failure of corner columns on the first floor of the structure (Column A-1). 2) Failure of the first floor long-side center columns (Column B-1). 3) Failure of the first floor short-side center columns (Column A-4), and 4) Failure of interior columns on the first floor of the structure (Column B-4).

### 3.4 Progressive collapse analysis considering corrosion

Once the simplified structural model is established, the corrosion damage of different materials (e.g., reinforcement, steel components) over various service periods is calculated. Based on the calculated damage and following the principles of continuum damage mechanics (e.g., Eq. (1)), key material properties such as yield strength and ultimate strength are reduced accordingly. These degraded material properties are then assigned to the service structural model, and using the approaches described in Sections 3.2 and 3.3, a time-dependent structural model can be constructed to perform progressive collapse analysis that accounts for corrosion-induced material degradation.


$$X=X_{0}\left(1-d\right)$$ (1)

Where *X* represents the residual mechanical properties of the material; *X*_0_ denotes the initial mechanical properties, and *d* is the damage index representing the degradation of material properties caused by corrosion.

## 4 Progressive collapse analysis of structure

### 4.1 Limit state definition of structure

**Fig 9:**
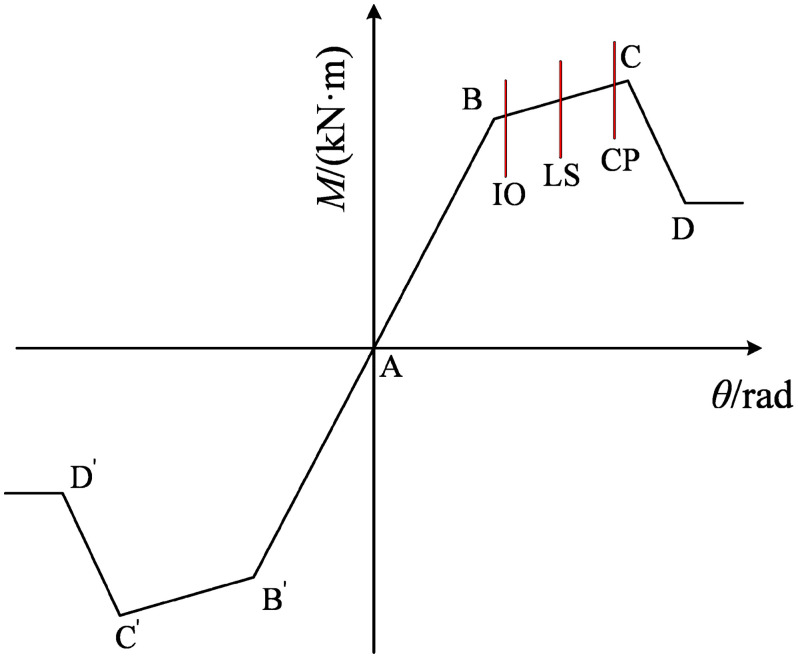
Bending moment and angle curve of plastic hinge. Note: Point A in this figure is the initial point; point B is the yield point, and point C is the ultimate bearing capacity; D point is the residual strength; IO, LS and CP represent direct use, life safety and collapse prevention respectively.

The progressive collapse analysis of structures typically involves significant nonlinear processes. During these processes, the structure transitions from the initial elastic stage to a nonlinear stage where it still retains the capacity to sustain external loads, until the structural demand exceeds its capacity, leading to progressive collapse. Therefore, it is essential to define the collapse limit state in advance when conducting progressive collapse resistance analysis. In this study, the collapse limit is determined based on the plastic hinge at the beam and column ends reaching the collapse prevention (CP) limit state (Fig 9), in accordance with the recommendations of FEMA 356 [31]. Alternatively, progressive collapse is considered to initiate when the displacement at the failure point reaches 1/10 of the beam span [32,33].

### 4.2 Dynamic analysis of the structure after column extraction

In the past, the structural progressive collapse analysis usually observed the internal force changes of the structure [34]. Therefore, this study takes the displacement response of the failure point and the axial force time history of the adjacent members as the evaluation indexes to assess the progressive collapse resistance of the PRCS and CPCS based on this. In the analysis process, the adjacent column closest to the failure column is selected as the research object. When there are multiple equidistant columns, the component with the largest force is preferentially selected. In this paper, the analysis time of PRCS and CPCS was set to 3 seconds, and the column removal operation is performed from 0.5 seconds under 1.0 times design load for comparative investigation.

**Fig 10:**
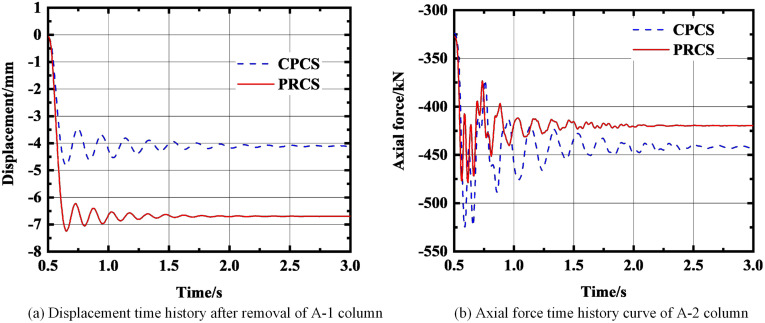
The force change of the structure after removing the A-1 column. **(a)** Displacement time history after removal of A-1 column. **(b)** Axial force time history curve of A-2 column.

**Fig 11:**
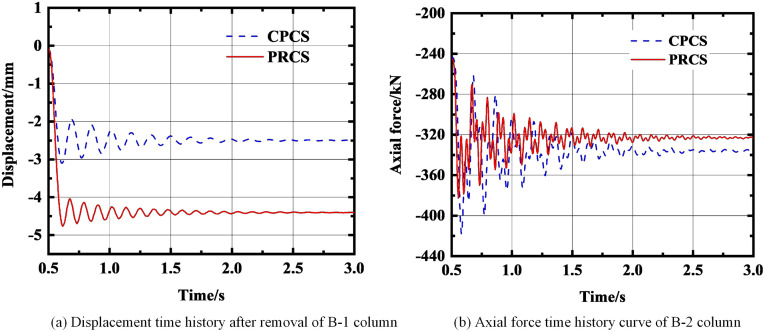
The force change of the structure after removing the B-1 column. **(a)** Displacement time history after removal of B-1 column. **(b)** Axial force time history curve of B-2 column.

**Fig 12:**
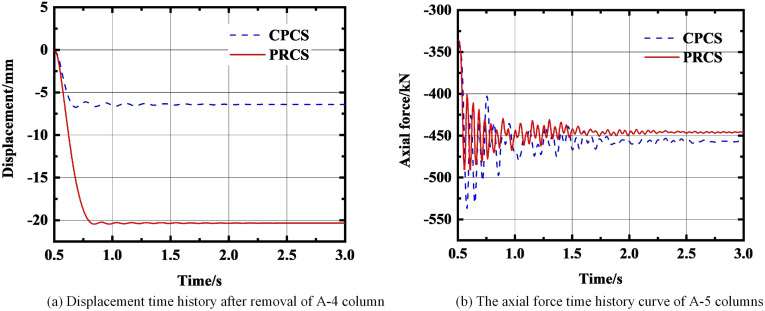
The force change of the structure after removing the A-4 column. **(a)** Displacement time history after removal of A-4 column. **(b)** The axial force time history curve of A-5 columns.

**Fig 13:**
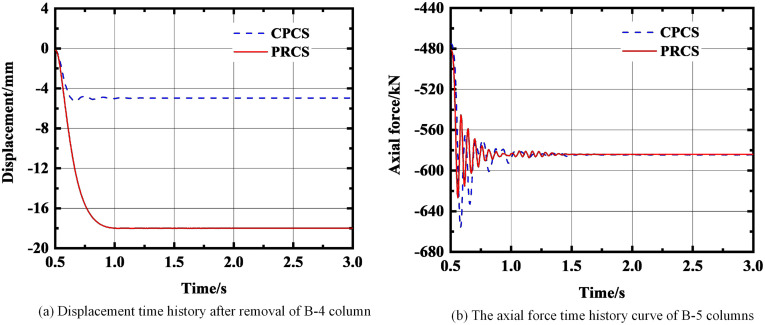
The force change of the structure after removing the B-4 column. **(a)** Displacement time history after removal of B-4 column. **(b)** The axial force time history curve of B-5 columns.

Through the comparative analysis of Figs 10–13, it can be seen that under the four typical working conditions, no progressive collapse occurred in the two types of structural systems. Specifically, in the case of A-1 column removal (Fig 10), the vertical displacement of the CPCS is finally stabilized at −4.1 mm, while that of the PRCS −6.7 mm, with an increase of 38.9%. For the axial force response, the axial force of the CPCS shows significant pre-oscillation characteristics, and the final stable value is −441.9 kN. In contrast, the axial force evolution curve of the PRCS is more stable, and its final value is −419.7 kN, which is 5% lower than that of the CPCS. The difference in displacement is primarily due to the three-dimensional stiffness of the PRCS frame being lower than that of the CPCS, which leads to larger deformation under the same column removal scenario. For the B-1 column removal condition (Fig 11), the final displacement of the CPCS and PRCS is −2.5 mm and −4.4 mm, respectively, and the displacement difference is 1.9 mm. The final values of the axial force are −336 kN and −323 kN, respectively. The axial force of the PRCS is reduced by 3.9% compared with the CPCS, as shown in Fig 11. For these two conditions, the mechanical properties of the PRCS and the CPCS are basically the same when the corner column and the long side column are removed. Therefore, the design method of the CPCS can be used in the progressive collapse design of the PRCS. The structural dynamic response after the final removal of the A-1 and B-1 column is summarized in Table 4.

**Table 4:** Structural response after removal of B-1 column.

Structural response	Removing the A-1 column		Removing the B-1 column	
	CPCS	PRCS	CPCS	PRCS
Displacement/mm	−4.1	−6.7	−2.49	−4.40
Axial force/kN	−441.9	−419.7	−336.59	−321.12
Total energy consumption	6.94*10^4	5.12*10^4	9.56*10^4	1.75*10^5

As shown in Fig 12, when the A-4 column is removed, the displacement of the CPCS is finally stabilized at −6.4 mm, and the displacement of the PRCS is finally stabilized at −20.3 mm. Under this working condition, the displacement of the PRCS and CPCS is quite different, and the displacement of the PRCS is 68.4% larger than that of the CPCS. For the axial force, the axial force of the CPCS still fluctuates greatly in the early stage. Finally, the axial force of the CPCS is stabilized at −456.9 kN, and the axial force of the PRCS is stabilized at −445.9 kN. The difference between the two axial forces is small. As shown in Fig 13, when the B-4 column is removed, the displacement of the CPCS is finally stabilized at −5 mm, and the displacement of the PRCS is finally stabilized at −18 mm. The displacement of the PRCS is 72.2% larger than that of the CPCS. At this time, the axial force of the PRCS and the CPCS is basically the same. According to the time-history displacement results, it is shown that under the condition of removing the short-side middle column and the inner column, when the initial failure of the component occurs, the PRCS will undergo large deformation. Although the prefabricated column connected by the outer steel tube bolt shows sufficient stiffness at the component level [20], there will still be insufficient stiffness in the three-dimensional space structure, which will cause large displacement of the structure and increase the risk of progressive collapse of the structure. Therefore, the cross-sectional dimensions of the side column and inner column of the PRCS and the thickness of the outer steel pipe should be appropriately increased for additional stiffness reserve. The structural dynamic response after the final removal of the A-4 and B-4 column is summarized in Table 5. Furthermore, based on the energy dissipation indicators in Tables 4 and 5, it can be observed that when prefabricated columns with steel-tube stud connections (PRCS) are employed within the overall frame, their energy dissipation capacity is, in most cases, significantly lower than that of CPCS structures. This may be attributed to the relatively limited ductility of the steel-tube stud connection nodes and the incomplete formation of local plastic hinges.

**Table 5:** Structural response after removal of B-4 column.

Structural response	Removing the A-4 column		Removing the B-4 column	
	CPCS	PRCS	CPCS	PRCS
Displacement/mm	−6.4	−20.3	−2.49	−5.00
Axial force/kN	−456.9	−445.9	−583.95	−581.12
Total energy consumption/J	9.45*10^4	1.75*10^4	4.29*10^5	2.59*10^5

### 4.3 Pushdown analysis of the structure

**Fig 14:**
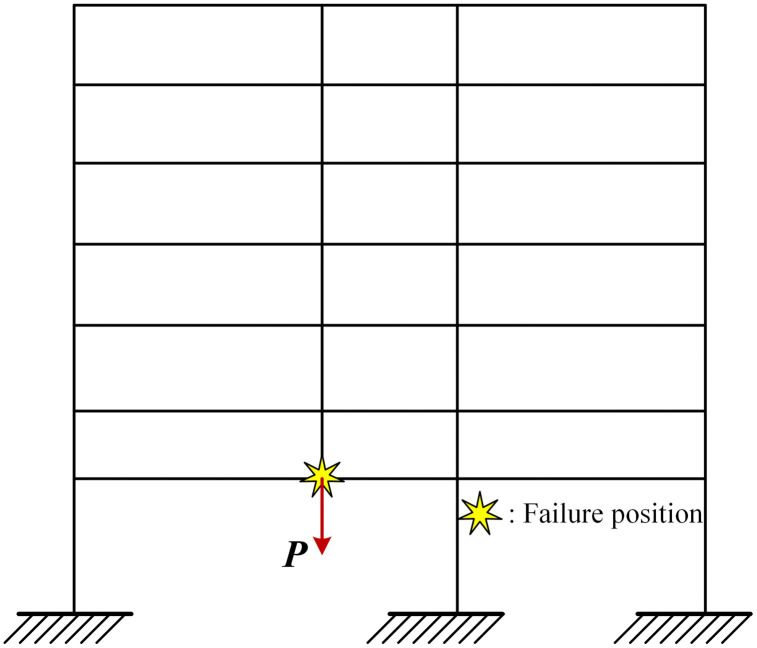
Pushdown loading diagram.

Based on the analysis results of the above column extraction method, it can be seen that there is still a certain difference in the collapse resistance between the PRCS and the CPCS. Therefore, in order to further clarify the difference between the collapse mechanism of the PRCS and the CPCS, the Pushdown analysis of the structure is further carried out. According to the different loading positions, Pushdown analysis can be divided into damaged span loading and full span loading. Lu et al.[34] studied the influence of Pushdown loading mode on the collapse resistance of the structure. The results show that the structural resistance curves under different loading modes are almost the same. Therefore, this paper uses damaged span loading, and the loading diagram is shown in Fig 14.

**Fig 15:**
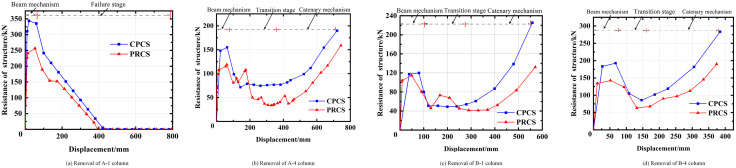
Pushdown curve of PRCS and CPCS. **(a)** Removal of A-1 column. **(b)** Removal of A-4 column. **(c)** Removal of B-1 column. **(d)** Removal of B-4 column.

From the Pushdown analysis results in Fig 15, it can be seen that when the four parts of the PRCS structure are removed respectively, the first peak bearing capacity appears when the displacement reaches 80 mm – 100 mm. This peak bearing capacity is mainly contributed by the collapse resistance of the beam mechanism of the structure, and its value is slightly lower than the collapse resistance of the beam mechanism of the CPCS. This is mainly because the local stiffness of the PRCS beam-column connections is lower, which delays the engagement of the catenary mechanism. When the A-1 column is removed (Fig 15a), whether it is an PRCS or CPCS, the structure quickly undergoes large displacement after reaching the bearing capacity of the beam mechanism. When the displacement reaches 400 m, both the PRCS and CPCS show progressive collapse. This is mainly due to the fact that after the corner column is removed, the beam loses its horizontal constraint and cannot provide enough axial force at the beam end, so that it cannot smoothly transition to the catenary mechanism, resulting in earlier damage. However, when the A-4, B-1 and B-4 columns are removed respectively, the plastic hinge of the beam end gradually begins to fail after the PRCS and the CPCS reach the bearing capacity of the beam mechanism, which makes the displacement of the structure increase rapidly. At the same time, the axial force of the beam end also increases rapidly, which further improves the resistance of the structure and makes the structure smoothly transform from the beam mechanism to the catenary mechanism. Among the four working conditions, regarding the load carrying capacity of the beam mechanism, the difference between the PRCS and the CPCS is the largest when column A-1 is removed, and the load carrying capacity of the beam mechanism of the CPCS is 25.4% higher than that of the PRCS; while the smallest difference is in the case of column B-1 removal, and the difference between the two is only 2.4%, as shown in Table 2.

Because the section height of the concrete beam in this paper is small and the reinforcement is relatively large, the bearing capacity of the catenary mechanism is slightly higher than that of the beam mechanism [25]. For the bearing capacity of the catenary mechanism, the largest gap between the PRCS and the CPCS is the removal of the B-4 working condition, and the bearing capacity of the catenary mechanism of the CPCS is 33.1% higher than that of the PRCS. The smallest difference is the case of removing the A-4 column, and the difference between the two is only 16.3%, as shown in Table 6. In summary, under the condition of removing A-4 column and B-1 column, the progressive collapse performance of the PRCS is close to that of the CPCS. However, under the condition of removing A-1 column and B-4 column (side column and inner column), the difference between the two is large. Therefore, in order to effectively prevent the occurrence of severe collapse, it is necessary to improve the cross-sectional dimensions and reinforcement of inner columns, side columns and their adjacent beam members.

**Table 6:** Comparison of bearing capacity of PRCS and CPCS under beam mechanism and catenary mechanism.

Conditions		*P*_CPCS_/kN	*P*_PRCS_/kN	(*P*_CPCS_ – *P*_PRCS_)/ *P*_PRCS_/%
Beam mechanism	Remove A-1 column	343.7	256.4	25.4
	Remove B-1 column	117.3	114.5	2.4
Catenary mechanism	Remove B-4 column	283.6	189.8	33.1
	Remove A-4 column	189.9	159.0	16.3

**Fig 16:**
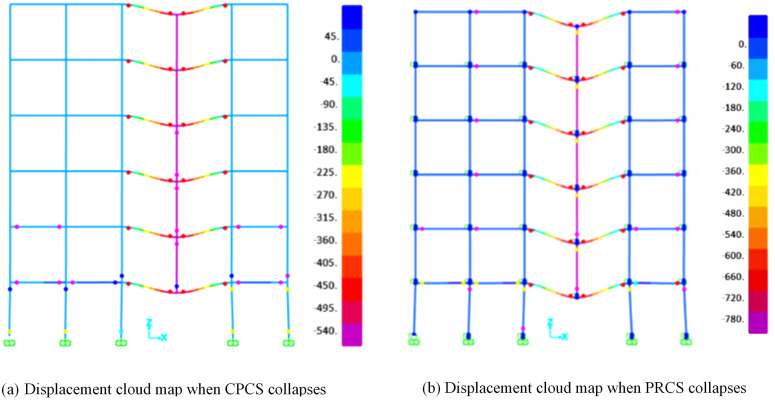
Plastic hinge distribution at collapse stage under B-4 column removal scenario. **(a)** Displacement cloud map when CPCS collapses. **(b)** Displacement cloud map when PRCS collapses.

In addition, as shown in Fig 16, the plastic hinge distribution under the column-removal scenario further illustrates the different collapse mechanisms between PRCS and CPCS. For the PRCS, more severe hinge development is observed in the first-story columns, and additional plastic hinges also occur in the upper-story members, indicating a faster degradation of load redistribution capacity compared to the CPCS. By contrast, the CPCS shows a relatively concentrated hinge formation in the lower-story columns and beams, while the upper structure remains less affected. Therefore, in practical applications, it is necessary to enhance the design grade of the first-story and upper-story key columns in PRCS to mitigate this weakness.

## 5 Pushdown analysis of structure considering corrosion damage

For concrete structures in coastal cities, they are usually subjected to long-term chloride corrosion damage during service, resulting in structural performance degradation. However, the connection joints of prefabricated concrete structure are usually composed of steel, and the anti-corrosion coating of steel is also prone to failure, which leads to corrosion of the connection device, reduces the safety performance of the structure, and increases the risk of progressive collapse of the structure. Therefore, this section further evaluates the life-cycle progressive collapse resistance of PRCS by considering the chloride corrosion damage.

### 5.1 Reinforcement corrosion initiation time

The corrosion degradation of chloride ions on rebar can generally be described by the classical Fick’s second law [35]. For example, Eq. (2) can accurately calculate the chloride ion concentration at different depths and different times of concrete:


$$C(x,t)=C_{0}\left[1-erf\left(\frac{x}{2\sqrt{k_{e}k_{t}k_{c}D_{0}{\left(t_{0}\right)}^{n}{\left(t\right)}^{1-n}}}\right)\right]$$ (2)

where *C*(*x*,*t*) is the chloride ion concentration at time *t* and *x* depth from the concrete surface; *C*_0_ is the chloride ion concentration on the surface of concrete; *erf* (·) is the error function; *k*_e_ is the environmental impact coefficient; *k*_t_ is the correction coefficient of test; *k*_c_ is the correction coefficient of curing condition; *D*_0_ is the chloride ion diffusion coefficient when the concrete age is *t*_0_; *n* is the service factor.

When the thickness of the concrete cover is *d*_c_, the initial corrosion time of the rebar can be solved according to Eq. (3) [36]:


$$T_{corr}={\left\{\frac{d_{c}^{2}}{4k_{e}k_{t}D_{0}{\left(t_{0}\right)}^{n}}{\left[erf^{-1}\left(\frac{C_{0}-C_{cr}}{C_{0}}\right)\right]}^{-2}\right\}}^{\frac{\text{1}}{1-n}}$$ (3)

where *T*_corr_ is the time of corrosion of rebar; *C*_cr_ is the critical chloride ion concentration for rebar corrosion.

### 5.2 Steel corrosion modeling

For the corrosion degradation model of steel, this paper uses the corrosion model obtained by Liang et al. [37] based on the 8-year and 16-year steel exposure tests conducted in China ‘s coastal environment:


$$D=At^{n1}$$ (4)

where *D* is the corrosion depth of steel; *t* is the service time; *a* and *n*_1_ are the steel type and the uncertainty environmental factor coefficient.

### 5.3 Quantification of corrosion damage

For the quantification of corrosion damage of rebar and steel, this paper quantifies corrosion damage based on the continuous damage mechanics, such as Eq. (5) [38].


$$d(t)=\frac{A(t)-A_{0}}{A(t)}$$ (5)

where *d*(*t*) is the corrosion damage at *t* time; *A*_0_ is the initial cross-sectional area of rebar or steel respectively; *A*(*t*) is the cross-sectional area of rebar or steel after service time *t*, respectively. The above area can also be converted to the mass reduction value after corrosion.

When the corrosion damage of rebar and steel is known, the residual mechanical properties of rebar and steel at any time can be estimated by using the residual performance calculation model provided by Du et al. [39] and Wu et al. [40] (such as Eqs. (6) and (7)).


$$X_{r}(t)=X_{0}^{\prime }(1-0.005d(t))$$ (6)

$$f_{y}(t)=f_{y0}(1-1.1115d(t))$$ (7-1)

$$f_{u}(t)=f_{u0}(1-0.9981d(t))$$ (7-2)

$$E(t)=E_{0}(1-1.1617d(t))$$ (7-3)

where *X*_r_(*t*) is the yield strength or ultimate strength after corrosion of rebar; *X′*_0_ is the yield strength or ultimate strength of uncorroded rebar. *f*_*y*_(*t*) is the yield strength of steel after corrosion; *f*_*y*0_ is the yield strength of uncorroded steel; *f*_u_(*t*) is the ultimate strength of steel after corrosion; *f*_u0_ is the ultimate strength of uncorroded steel; *E*(*t*) is the elastic modulus of steel after corrosion; *E*_0_ is the elastic modulus of uncorroded steel. After reducing the key material parameters of the structure (such as yield strength and ultimate strength) using Eqs. (6) and (7), the degraded material properties can be assigned to the numerical model to analyze the progressive collapse performance of the structure at that stage.

It is worth noting that the corrosion damage will also lead to the degradation of the constraint rebar, which will reduce the performance of the concrete in the core area. In this case, Mander concrete constraint constitutive is used to consider the influence of environmental factors on its performance degradation. For this example, the residual strength of reinforcement and steel under different corrosion damage (i.e., different service time) is shown in Table 7.

**Table 7:** Residual yield strength of materials under different corrosion damage.

Materials	T = 0	T = 20	T = 40	T = 60
Reinforcement	490	454.26	414.07	315.19
Steel	320	301.97	269.01	226.33

### 5.4 Structural resistance curves considering corrosion damage

**Fig 17:**
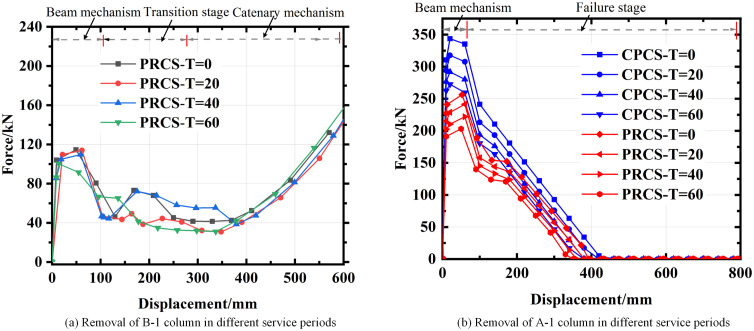
Pushdown curve evolution process of structure considering corrosion damage. **(a)** Removal of B-1 column in different service periods. **(b)** Removal of A-1 column in different service periods.

It can be seen from Fig 17(a) that when the B-1 column (short-side middle column) is removed, the effect of corrosion on the progressive collapse resistance is limited at the early stage, as the beam mechanism still provides sufficient load redistribution. However, with the accumulation of corrosion damage, the beam mechanism of PRCS rapidly loses its residual capacity, leading to a lack of reserve strength. This prevents the structure from developing an effective catenary action, which is critical for maintaining stability after the beam mechanism fails. Consequently, the structure is more likely to collapse during the small-displacement stage, resulting in a sudden loss of load-bearing capacity and potentially causing severe casualties.

For the A-1 column (corner column) removal scenario shown in Fig 17(b), corrosion significantly reduces the ultimate displacement capacity of the PRCS. Compared with the short-side middle column removal case, the corner column removal case exhibits stronger brittleness, as the structural system relies more on the catenary action of the surrounding beams. Corrosion may weaken both the reinforcement and the bond performance, accelerating brittle fracture and inhibiting ductile redistribution.

Therefore, corrosion has different impacts depending on the failure mode: in the short-side middle column removal case, it mainly reduces the reserve capacity of the beam mechanism, while in the corner column removal case, it promotes brittle failure and reduces the overall ductility. These results highlight the necessity of considering long-term corrosion effects in PRCS design, such as increasing the thickness of the concrete cover layer to delay corrosion initiation, or adopting high-strength reinforcement to maintain sufficient residual strength.

## 6 Conclusions

In this paper, the model based on SAP2000 finite element software is verified by the test of three prefabricated columns with stud connection. The results show that the simulated values are basically consistent with the experimental values. On this basis, the collapse resistance of a 6-story PRCS and CPCS was studied. The main conclusions are as follows:

1) The simplified model of prefabricated column based on multi-segment linear plastic connection element has good calculation accuracy and can be effectively used for the analysis of progressive collapse resistance of structures. The error between the test value and the simulated value is less than 7%.
2) When the short-side middle column and the inner column are removed, the PRCS exhibits insufficient stiffness compared with the CPCS under the same reinforcement ratio, as the maximum displacement of the PRCS increases by up to 68.4% relative to the CPCS. Therefore, it is necessary to increase the cross-sectional dimensions of the PRCS short-side center column and inner column, as well as the thickness of the outer steel tube, to enhance structural stiffness
3) When the corner column is removed, the bearing capacity of the PRCS decreases significantly compared with the CPCS, with the beam mechanism capacity of the PRCS being 25.4% lower than that of the CPCS. In contrast, when the long-side middle column is removed, the beam mechanism capacities of the PRCS and CPCS are comparable, differing by only 2.4%. These results indicate that the design grade of the corner column should be enhanced for the PRCS to improve its progressive collapse resistance.
4) The pushdown analysis further confirms that PRCS exhibits lower catenary action and ultimate displacement capacity than CPCS, particularly when inner column or corner column is removed.
5) Under specific scenarios such as the long-side middle column removal, the collapse resistance of PRCS is generally comparable to CPCS, suggesting that PRCS retains a favorable capacity to sustain progressive collapse despite its modular nature.
6) Long-term corrosion damage will further increase the possibility of progressive collapse of PRCS, which needs to be considered in design process. For example, design measures such as increasing the thickness of the concrete cover and using high-strength rebar can be adopted to mitigate the adverse effects of corrosion.

Because this paper only discusses the progressive collapse behavior of PRCS under four working conditions, and does not fully consider the influence of the uncertainty of structure and chloride corrosion damage, or the dynamic effects captured by the Dynamic Increase Factor (DIF) on the progressive collapse performance. Therefore, there are still some limitations in the assessment of the progressive collapse performance of the life cycle of the structure. In the future research, it is necessary to further explore the modeling under various working conditions, considering corrosion and structural uncertainty, and establishing corresponding progressive collapse performance evaluation methods.

## Supporting information

S1 File Data on manuscripts. 1. Raw experimental and simulated load-displacement data for all specimens presented in Fig 7. 2. Time-displacement data for dynamic analysis presented in Fig 10. 3. Time-displacement data for dynamic analysis presented in Fig 11. 4. Time-displacement data for dynamic analysis presented in Fig 12. 5. Time-displacement data for dynamic analysis presented in Fig 13. 6. Force-displacement data for progressive collapse analysis under different column removal scenarios presented in Fig 15. 7. Force-displacement data at different time points for structural response presented in Fig 17.
(DOCX)
