# Supplementary material for: Finite element analysis of progressive collapse resistance of a prefabricated RC frame structure with stud connection considering chloride corrosion
Source: PLoS One. 2025 Nov 20;20(11):e0333741. doi: 10.1371/journal.pone.0333741 (PMC12633900; doi:10.1371/journal.pone.0333741)
Supplement: S1 File — 1. Raw experimental and simulated load-displacement data for all specimens presented in Fig 7. 2. Time-displacement data for dynamic analysis presented in Fig 10. 3. Time-displacement data for dynamic analysis presented in Fig 11. 4. Time-displacement data for dynamic analysis presented in Fig 12. 5. Time-displacement data for dynamic analysis presented in Fig 13. 6. Force-displacement data for progressive collapse analysis under different column removal scenarios presented in Fig 15. 7. Force-displacement data at different time points for structural response presented in Fig 17. (DOCX) [file pone.0333741.s001.docx]

**S1 Data. Raw experimental and simulated load-displacement data for all specimens presented in Fig 7.**
This spreadsheet contains the complete set of numerical data used to generate the load-displacement curves in Figure 7 of the main text. The data for all four specimens (PRCC-W01, W02, W03, W04) is consolidated within the "Sheet01". For each specimen, the data is structured as follows:

- **PRCC-W01:** Column A: Experimental Displacement (mm); Column B: Experimental Load (kN); Column C: Simulated Displacement (mm); Column D: Simulated Load (kN).
- **PRCC-W02:** Column E: Experimental Displacement (mm); Column F: Experimental Load (kN); Column G: Simulated Displacement (mm); Column H: Simulated Load (kN).
- **PRCC-W03:** Column I: Experimental Displacement (mm); Column J: Experimental Load (kN); Column K: Simulated Displacement (mm); Column L: Simulated Load (kN).
- **PRCC-W04:** Column M: Experimental Displacement (mm); Column N: Experimental Load (kN); Column O: Simulated Displacement (mm); Column P: Simulated Load (kN).

The experimental data (plotted in black in Fig 7) were measured during laboratory tests, while the simulated data (plotted in red in Fig 7) were outputs from the finite element analysis.

**S1 Data. Time-displacement data for dynamic analysis presented in Fig 10.**

This spreadsheet contains the numerical data used to generate the time-displacement curves in Figure 10 of the main text. The data is organized into two main sections, corresponding to each subfigure:

- **For Fig 10a:** This section contains time-displacement data for the CPCS and PRCS models under a global dynamic analysis.
  - **CPCS Model:** Column A: Time (s); Column B: Displacement (mm).
  - **PRCS Model:** Column C: Time (s); Column D: Displacement (mm).
- **For Fig 10b:** This section contains time-displacement data for the CPCs and PRCS models under a global dynamic analysis.
  - **CPCS Model:** Column E: Time (s); Column F: Displacement (mm).
  - **PRCS Model:** Column G: Time (s); Column H: Displacement (mm).

**S1 Data. Time-displacement data for dynamic analysis presented in Fig 11.**

This spreadsheet contains the numerical data used to generate the time-displacement curves in Figure 11 of the main text. The data is organized into two main sections, corresponding to each subfigure:

- **For Fig 11a:** This section contains time-displacement data for the CPCS and PRCS models under a global dynamic analysis.
  - **CPCS Model:** Column A: Time (s); Column B: Displacement (mm).
  - **PRCS Model:** Column C: Time (s); Column D: Displacement (mm).
- **For Fig 11b:** This section contains time-displacement data for the CPCs and PRCS models under a global dynamic analysis.
  - **CPCS Model:** Column E: Time (s); Column F: Displacement (mm).
  - **PRCS Model:** Column G: Time (s); Column H: Displacement (mm).

**S1 Data. Time-displacement data for dynamic analysis presented in Fig 12.**

This spreadsheet contains the numerical data used to generate the time-displacement curves in Figure 12 of the main text. The data is organized into two main sections, corresponding to each subfigure:

- **For Fig 12a:** This section contains time-displacement data for the CPCS and PRCS models under a global dynamic analysis.
  - **CPCS Model:** Column A: Time (s); Column B: Displacement (mm).
  - **PRCS Model:** Column C: Time (s); Column D: Displacement (mm).
- **For Fig 12b:** This section contains time-displacement data for the CPCs and PRCS models under a global dynamic analysis.
  - **CPCS Model:** Column E: Time (s); Column F: Displacement (mm).
  - **PRCS Model:** Column G: Time (s); Column H: Displacement (mm).

**S1 Data. Time-displacement data for dynamic analysis presented in Fig 13.**

This spreadsheet contains the numerical data used to generate the time-displacement curves in Figure 13 of the main text. The data is organized into two main sections, corresponding to each subfigure:

- **For Fig 13a:** This section contains time-displacement data for the CPCS and PRCS models under a global dynamic analysis.
  - **CPCS Model:** Column A: Time (s); Column B: Displacement (mm).
  - **PRCS Model:** Column C: Time (s); Column D: Displacement (mm).
- **For Fig 13b:** This section contains time-displacement data for the CPCs and PRCS models under a global dynamic analysis.
  - **CPCS Model:** Column E: Time (s); Column F: Displacement (mm).
  - **PRCS Model:** Column G: Time (s); Column H: Displacement (mm).

**S1 Data. Force-displacement data for progressive collapse analysis under different column removal scenarios presented in Fig 15.**

This spreadsheet contains the numerical data used to generate the force-displacement curves in Figure 15 of the main text, which compares the progressive collapse resistance of the CPCS (blue) and PRCS (red) models. The data is organized into four sections, corresponding to each subfigure and column removal scenario:

- **For Fig 15a (Removal of A-1 column):** This section contains force-displacement data for the CPCS and PRCS models.
  - **CPCS Model (blue):** Column A: Displacement (mm); Column B: Force (kN).
  - **PRCS Model (red):** Column C: Displacement (mm); Column D: Force (kN).
- **For Fig 15b (Removal of A-4 column):** This section contains force-displacement data for the CPCS and PRCS models.
  - **CPCS Model (blue):** Column E: Displacement (mm); Column F: Force (kN).
  - **PRCS Model (red):** Column G: Displacement (mm); Column H: Force (kN).
- **For Fig 15c (Removal of B-1 column):** This section contains force-displacement data for the CPCS and PRCS models.
  - **CPCS Model (blue):** Column I: Displacement (mm); Column J: Force (kN).
  - **PRCS Model (red):** Column K: Displacement (mm); Column L: Force (kN).
- **For Fig 15d (Removal of B-4 column):** This section contains force-displacement data for the CPCS and PRCS models.
  - **CPCS Model (blue):** Column M: Displacement (mm); Column N: Force (kN).
  - **PRCS Model (red):** Column O: Displacement (mm); Column P: Force (kN).

The data demonstrates the structural response and comparative performance of the two models in resisting progressive collapse under various critical scenarios, as determined by finite element analysis.

**S1 Data. Force-displacement data at different time points for structural response presented in Fig 17.**

This spreadsheet contains the numerical data used to generate the force-displacement curves in Figure 17 of the main text, which tracks the mechanical response of the PRCS and CPCS models over time.

- **PRCS Model (Fig.17a):** This dataset captures the structural response of the PRCS model at four distinct time points.
  - **T=0:** Column A: Displacement (mm); Column B: Force (kN).
  - **T=20:** Column C: Displacement (mm); Column D: Force (kN).
  - **T=40:** Column E: Displacement (mm); Column F: Force (kN).
  - **T=60:** Column G: Displacement (mm); Column H: Force (kN).
- **CPCS Model (Fig.17b):** This dataset captures the structural response of the CPCS model at four distinct time points.
  - **T=0:** Column L: Displacement (mm); Column M: Force (kN).
  - **T=20:** Column N: Displacement (mm); Column O: Force (kN).
  - **T=40:** Column P: Displacement (mm); Column Q: Force (kN).
  - **T=60:** Column R: Displacement (mm); Column S: Force (kN).
- **PRCS Model (Fig.17b):** This dataset captures the structural response of the PRCS model at four distinct time points.
  - **T=0:** Column T: Displacement (mm); Column U: Force (kN).
  - **T=20:** Column V: Displacement (mm); Column W: Force (kN).
  - **T=40:** Column X: Displacement (mm); Column Y: Force (kN).
  - **T=60:** Column Z: Displacement (mm); Column AA: Force (kN).

The data illustrates the evolution and potential degradation of stiffness and strength in the two structural models over the specified time period, as predicted by the finite element analysis.
